# A toolkit for publishing enhanced figures

**DOI:** 10.1107/S0021889808015616

**Published:** 2008-07-01

**Authors:** Brian McMahon, Robert M. Hanson

**Affiliations:** a International Union of Crystallography, 5 Abbey Square, Chester CH1 2HU, UK; b Department of Chemistry, St Olaf College, Northfield, MN 55057, USA

**Keywords:** computer programs, interactive graphics, molecular visualization

## Abstract

An editing toolkit has been developed to allow authors to create enhanced interactive figures for publication in crystallography journals. The visualization engine is *Jmol*, a Java program that can be manipulated as an applet in a web browser. The toolkit provides user-friendly widgets to manipulate the graphics state of *Jmol*, and persistent storage of graphics state, scripts and associated data files.

## Abstract 2

A description is provided of a software utility for creating interactive figures derived from crystal structures using the Java program *Jmol*.

## 1. Introduction

The use of molecular graphics visualization software in association with journal articles reporting or describing crystal and molecular structures is not new.

In 1993, the journal *Protein Science* published a CD-ROM containing the entire contents of its first volume of publication, the entire contents of the Protein Data Bank, and copies of *MAGE* and *PREKIN* software for creating and viewing ‘kinemages’, annotated interactive views of three-dimensional protein structures (Richardson & Richardson, 1992 ▶).

In the mid-1990s, *Chemical Communications* acted as a test bed for a variety of new web technologies supporting enhanced and interactive figures, implemented by the CLIC Consortium (Murray-Rust *et al.*, 1997 ▶).

**Figure 1:**
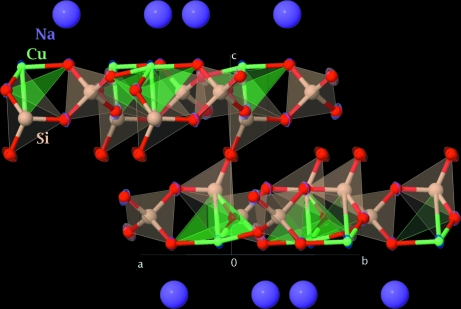
View of the crystal structure of disodium dicopper undecaoxide tetrasilicate (Cunha-Silva *et al.*, 2008 ▶), highlighting recent visualization enhancements in *Jmol*.

More recently, *Biochemical Journal* and *ACS Chemical Biology* have hosted a number of papers with interactive figures and animations as supporting materials [*e.g.* Himmel *et al.* (2006 ▶), for which the supplementary figures may be viewed at http://pubs.acs.org/journals/acbcct/weos/ACSviewer/0612himmel/figure1.htm].

While these initiatives have all added value to the scientific articles in which they have been involved, they have been experimental prototypes, have depended on specific software or operating system requirements that have not been supported over the passage of time, or have provided supplementary information, not the main content of an article.

With the maturing of web standards and the gradual movement towards electronic only journal publication, the IUCr has developed a toolkit for authors in its journals to produce enhanced interactive figures that form an integral part of the article and that are handled efficiently in the peer review and editorial production systems (Fig. 1 ▶).

## 2. *Jmol* as the graphics engine

The enhanced figure toolkit (*jtkt*) provides an environment for creating visualizations of crystal and molecular structures using the program *Jmol* (2008 ▶). *Jmol* is a highly respected program, the development of which has been influenced by the previously widespread chemical visualization tools *RasMol* (Sayle & Milner-White, 1995 ▶) and *Chime* (http://www.mdl.com/products/framework/chime/), among others. It has a number of features that make it attractive as the graphics engine for journal enhanced figures, including some characteristics that are essential to the working of the toolkit that we have developed.

The features that are attractive, but which in principle could be offered by other first-rate visualization programs currently available to the community, are

(i) support for a wide range of input file formats (including CIF and mmCIF)

(ii) support for common styles of representing inorganic, small-molecule and biological macromolecular structures

(iii) a wide range of colour schemes and labelling conventions

(iv) robust support for crystallographic symmetry operations

(v) the ability to display atomic displacement ellipsoids

The features that we consider essential for the use of such a program in an electronic journal production environment are the following:

(i) cross-platform compatibility

(ii) the ability to run the program as an embedded applet in a browser and to run it as a standalone application with command-line parameters

(iii) the availability of JavaScript libraries allowing communication with the application through buttons and checkboxes on a web page

(iv) the availability of source code through an open licence and an active developer community

(v) high-quality rendering of atoms, bonds and molecular surfaces, both interactively and exportable as *POV-Ray* (http://www.povray.org/) files or images

(vi) a fast, intuitive interactive model manipulation capability, including scripting, with help documentation for non-expert users

(vii) efficient export of the graphics state allowing all the properties of a particular view to be transmitted to other instances of the program

## 3. Philosophy behind ‘enhanced figures’

Interactivity allows a reader unbounded scope to explore a structure, taking advantage of whatever features the visualization software may provide. This is the basis underlying *FirstGlance in Jmol* (http://firstglance.jmol.org), a service that an increasing number of journals link to, which provides standard buttons to view different aspects of a protein structure as well as access through an in-applet menu to all of *Jmol*’s functions.

In a research article, however, the purpose of a figure is usually to draw the reader’s attention to specific features of the structure that the author has identified or singled out for particular scrutiny. An enhanced figure should preserve that purpose and allow the author to designate a preferred view of the structure with suitable annotations. Ideally, the author should also be able to provide the reader with specific options to view different selected aspects of the structure. None of this should interfere with the reader’s ability to explore the structure beyond the author’s preferred view, but the reader should always be able to return to the preferred view after experimentation. The reader may interrogate the figure for particular information (*e.g.* atom labels, symmetry operations, bond distances or angles).

In all respects, *Jmol* (and, to our knowledge, no other program) suits these needs. By virtue of being Java-based and coded with flexibility in mind, *Jmol* has broad cross-platform compatibility. No graphical libraries are used that would require installation or special hardware. Available as both Java application and Java applet, *Jmol* provides both application-specific capabilities (such as file reading from remote databases and file writing) and browser-independent functionality. Thus, *Jmol* views created by an author using the applet can be processed by the server using the *Jmol* application for the production of high-resolution PNG or JPEG images for use in publication or delivery back to the author. In addition, specifically for the applet, *Jmol* includes a JavaScript library that hides cross-browser compatibility issues from the web page developer and makes it very easy to customize views with buttons, checkboxes, selection boxes and HTML links. *Jmol*’s interface and scripting functionality have been well tested by hundreds of users, with extensive input from the crystallographic community. Online help, including extensive scripting examples, is readily available. Finally, recent versions of *Jmol* allow for the saving and restoring of the current state of the model in the form of script files. These scripts efficiently describe the exact state of the model and are not simply a historical list of commands given.

Publishers of research articles have the responsibility of archiving the record of science, so that the information in the figure must be capable of being stored and made accessible to readers in the long term, in spite of the changing landscape of visualization software. While no software can guarantee that the results can be archived in a readable form indefinitely, the use of open-source Java software in standards-compliant web browsers does make a material contribution in this direction. To address the archival issues we adopt a complementary approach; when the author saves a view in *Jmol*, a static image in TIFF format is exported showing the same initial view. Strictly, it is this static image (which, incidentally, is used in the PDF and printed editions of the journal article) that constitutes the archived figure.

This rather conservative approach explains the terminology ‘enhanced figure’. While the interactive elements of the structural model presented by the *Jmol* applet provide the reader with additional insight and value, these are considered extensions – enhancements – to the static view and not a replacement. This approach also means that readers without access to Java or JavaScript in their browsing environment can follow the scientific argument using the static figure (which is automatically provided if the browser cannot support the applet).

## 4. Architecture of the toolkit

The toolkit has server and client components. It is implemented as a single Perl module, Jtkt.pm, that operates as a mod_perl content handler in an Apache web server. This allows it to receive input both from the URL under which it is called (any path components after the base URL http://submission.iucr.org/jtkt are parsed as directives to the server) and through information in HTML forms passed through normal GET or POST HTTP methods by the client web browser.

### 4.1. Server-side features

The server component has a number of roles:

(i) It delivers an instance of the client process to the user – *i.e.* it generates the editing web page with forms, widgets, the *Jmol* applet and an initial *Jmol* loading script tailored to the type of structure being handled.

(ii) It provides and manages persistent storage of the data generated in a client session. Since the Web uses stateless protocols, the various scripts generated by the author during an editing session must be stored on the server machine and used to repopulate the browser page when a new session is initiated. The author’s CIF, which provides the structural data for the visualization, is also stored on the server.

(iii) It integrates the handling of the enhanced figure with the journal submission and production systems. Authors may create an enhanced figure before submitting their article or afterwards. Allocation of file storage, identification of the material and authentication of the user are all handled differently in these two cases, but the system must allow easy transfer from a pre-submission enhanced figure to the author’s submission area and subsequently to the journal editorial workspace. Nothing complicated is involved; it is mostly a matter of filesystem housekeeping and management. Nevertheless, performing these manipulations automatically is key to making the whole process possible within a journal production workflow.

(iv) It creates high-resolution static images in synchronization with the preferred initial view presented by the interactive applet.

### 4.2. Client-side features

The client side of the toolkit is seen by the user as a sequence of web pages or forms. The initial form allows the user to upload the CIF to the server and to provide an indication of the type of structure (which is used by the server to tailor the configuration of editing panels in subsequent pages).

**Figure 2:**
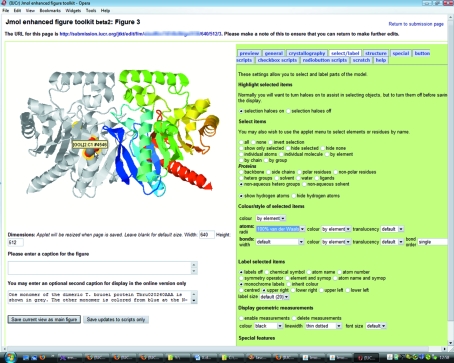
User interface to the enhanced figure editing toolkit. In this view, the user is highlighting a ligand group using the available options for selecting and labelling parts of the structure. The tabs provide access to different sets of options to implement various features offered by the *Jmol* visualizer. The structure is of a *Trypanosoma brucei* 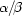-hydrolase-fold protein with unknown function (Merritt *et al.*, 2008 ▶).

Subsequently, the client application is characterized by a web page that includes the *Jmol* applet, various forms containing figure captions, information about the applet configuration and the contents of *Jmol* scripts that the author wishes to provide for the use of the reader in the final enhanced figure (Fig. 2 ▶).

#### 4.2.1. Editing tabs

The user workspace uses a tabbed interface to allow the author to concentrate on specific aspects of modifying the enhanced figure. For example, the **general** tab provides options for global choices, such as overall style (ball and stick, ellipsoids, macromolecular structure cartoons *etc.*), colour scheme, background colour, perspective view and stereo representation. The **crystallography** tab provides options for displaying the unit cell, extended crystal packing, highlighting of symmetry operations and so on. The **ellipsoid** tab provides options to change the size, style and colour of displacement ellipsoids; and so on.

*Jmol* has a very rich command language, and consequently a certain amount of experience is necessary to use it to its fullest capabilities. In providing focused collections of specific sets of *Jmol* operations, the intention is to help new *Jmol* users to discover and use the available functions and to guide them into creating visual representations that are close to journal style rules.

However, it is also possible for advanced users to communicate directly with *Jmol* through a pop-up console window, and so the creative author is not restricted by the selection of functions provided through the tabbed interface.

The various button, checkbox and menu widgets on these editing tabs are generated from the Jmol.js JavaScript library that is included in the *Jmol* distribution. They communicate directly with the *Jmol* applet to implement the scripts bound to each widget. This communication depends upon LiveConnect technology, which is not implemented in all browsers, although fortunately it is implemented in many of the browsers that are currently most widespread (Firefox, Safari, Opera, Internet Explorer).

#### 4.2.2. Script management tabs

A second group of tabs allows the author to insert additional *Jmol* scripts and associated rubrics that will be bound to individual button or checkbox widgets in the published enhanced figure. In this way the author can provide the reader with the ability to run specific scripts designed to illustrate particular aspects of the structure.

The author-supplied scripts are grouped as ‘button scripts’, single scripts, possibly complex in operation, which are run when the reader selects the associated button; ‘checkbox scripts’, which provide a box that allows the reader to toggle between two scripts, usually short and with opposing actions (*e.g.* turning a stereoview on or off); and ‘radiobutton scripts’, which typically allow the reader to select between alternative choices for a particular aspect of the structure (*e.g.* showing displacement ellipsoids at 30, 50 or 90%).

In fact any script may be bound to any of the available widgets, so the usage of button, checkbox or radiobutton as described is not mandatory, but authors are encouraged to use the widgets in this way to accommodate the reader’s normal expectation. The toolkit currently provides up to four ‘run-once’ buttons, six dual-state checkboxes and two groups of up to six radiobuttons, making a total of 24 possible ‘enhanced views’.

JavaScript functions are provided that allow import of the current graphics state of the *Jmol* applet into any of the author-generated scripts. This means that the author can use the editing tabs to create a new representation of the structure in the *Jmol* applet, of any level of complexity, and import the result as a complete script. This will prove particularly helpful to authors who are not familiar with *Jmol*’s scripting language in detail.

Other tabs allow the author to preview the action of any such scripts before the result is saved back to the server.

## 5. Documentation and availability

*Jmol* is available for download from http://www.jmol.org, and it has an extensive collection of help documentation at http://chemapps.stolaf.edu/jmol/docs. The full set of capabilities needed for implementing the system described in this article is found in *Jmol* versions 11.5.37 or later.

A user manual for the enhanced figure toolkit is available from the IUCr (http://journals.iucr.org/services/jtkt_manual.pdf). The source code contains much that arises from the need for tight integration with the journal production systems and is therefore not suitable for general distribution. However, a copy may be obtained upon application to the correspondence author. A version of the graphical editor is under development for release within the next version of *publCIF* (Westrip, 2008 ▶).

## Supplementary Material

Enhanced figure: interactive version of Fig. 1
